# Acupuncture for chronic pelvic inflammatory disease: a qualitative study of patients’ insistence on treatment

**DOI:** 10.1186/1472-6882-14-345

**Published:** 2014-09-21

**Authors:** Yudan Liang, Dongfang Gong

**Affiliations:** Guangzhou University of Chinese Medicine, 12 Ji Chang Rd, Guangzhou, China; Department of Acupuncture, 1st Affiliated Hospital, Guangzhou University of Chinese Medicine, 12 Ji Chang Rd, Guangzhou, China

**Keywords:** Acupuncture, Pelvic inflammatory disease, Qualitative research, Semi-structure interviews

## Abstract

**Background:**

Acupuncture is an effective complement to pharmacological therapy in the alleviation of chronic pelvic inflammatory disease (PID). It has mild or no side effects; however, a minimum of 3 months of therapy is required to guarantee a beneficial outcome. This study investigates why patients insist on acupuncture therapy to aid recuperation.

**Methods:**

The study included a purposive sample of 15 participants diagnosed with chronic PID who had received a course of acupuncture therapy at least twice a week for a minimum of 3 months. Semi-structured interviews were conducted, recorded, transcribed, coded and analyzed using systematic text condensation.

**Results:**

Four overarching themes were identified from the participants’ reasons for insisting on lengthy, but in their view important, acupuncture courses. The four overarching themes were: (1) the patients’ characteristics, including pregnancy aspiration and the fear of serious gynecological disease; (2) the patient–practitioner relationship, including the acupuncturist’s attitude towards the patients and the explanation of the disease from a traditional Chinese medicine (TCM) perspective; (3) the characteristics of acupuncture, including the diversification of treatment modes, the synthetical effect, and no side-effects; and (4) the clinical environment, including the exchange of experience between patients and the well-equipped setting.

**Conclusions:**

There were mixed reasons for patients diagnosed with chronic PID maintaining acupuncture treatments. Knowledge and understanding about the acupuncture-disease relationship were conducive to the patients’ preference for acupuncture. Acupuncture as a complement to Western medicine should be further developed while maintaining these positive features. Participants reported feeling hope, confidence, and a sense of responsibility for their treatment during the process, although the treatments did not always have the expected outcome.

## Background

Chronic pelvic inflammatory disease (PID) is associated with lower abdominal pain, low fever, irregular menstruation, and dysmenorrhea, is a leading cause of infertility and ectopic pregnancy, and is related to an increased risk of ovarian borderline tumor [1]. Patients with chronic PID often suffer from acute attack or immune deficiency, and inappropriate antibiotic management may result in endometritis, oophoritis, tubo-ovarian abscess or peritonitis [2]. Owing to the disease’s complex mechanism and long-term process, its definitive medical effect is weakened by side effects. Recent clinical and experimental studies on acupuncture for the treatment of chronic PID indicate that it can help improve the clinical symptoms and pregnancy rate [3–7]. Acupuncture exhibits anti-inflammatory and immunity-enhancing effects in women with PID, with significant drops in erythrocyte sedimentation rate (ESR) and immunoglobulin M (IgM) levels, as well as a rise in γ-globulin levels and a significant decrease in pain scores (from 4.89 ± 0.82 to 0.63 ± 1.05) [8]. Within a trial of acupuncture for chronic pelvic pain, 29 of the 30 patients experienced a clinical response to pelvic pain, and seven infertile patients became pregnant in the following 6 months [2].

Acupuncture may hence complement pharmacological therapy in the alleviation of chronic PID symptoms. Previous studies on the use of acupuncture for the treatment of knee osteoarthritis, polycystic ovary syndrome, menopausal hot flashes, and low back pain have been described qualitatively from the aspect of patient acceptability or preconception [9–13]. However, there is little information on the physical experiences of women with chronic PID treated with acupuncture. To ensure a long-term effective outcome from acupuncture, clinical experience shows that the course of therapy should be a minimum of 3 months [3, 5]. We therefore established a study to investigate the insistence by women with chronic PID on undergoing acupuncture to regain health. As a skillful clinical acupuncturist with a license for over 30 years, the second author (Gong) is adept at the treatment of gynecological diseases. The sample patients received traditional acupuncture performed by Gong at the First Hospital Affiliated with Guangzhou University of Traditional Chinese Medicine (TCM) in China.

## Methods

To investigate why patients insist on a long-term course of acupuncture, it is appropriate to use qualitative methods. This study complied with the Helsinki Declaration and was approved by the Ethics Committee of the First Affiliated Hospital, Guangzhou University of Chinese Medicine, China.

Patients were recruited through a recruitment poster on an outpatient notice board, and from 2013 computer medical records (contacted by telephone). Eligibility was based on the following criteria: (a) a history of chronic PID [3], (b) a course of acupuncture (with no concomitant treatment) at least twice a week for a minimum of 3 months, and (c) traditional acupuncture conducted by Gong. From these, a purposive sample was made of 15 participants for inclusion in the study. Written consent received relating to the context of the study was obtained, and an audio-recorded interview with all names replaced with pseudonyms was conducted at the time of interview.

Semi-structured interviews were conducted by the first author, who is trained in conducting qualitative studies and interviews. In face-to-face and one-on-one open interviews (without field notes) that were based on a prepared topic guide (Table 1) designed to draw out the experience accounts, the participants were able to elaborate freely and in comfort. Iterative questioning was necessary to establish credibility and trustworthiness with the participants. The interviews lasted approximately 30 minutes and were recorded, transcribed verbatim and checked for accuracy.

**Table 1:** **Main topic guide**

Description	Question(s)
Main question	I’d like to make a thorough investigation about the reasons of patients’ insistence on the long-term course of acupuncture treatment. Could you please tell me all about it?
Supplementary questions	How do you feel about your experience of acupuncture?
	Could you tell me about the opinion of acupuncturist?
	What is the different between acupuncture and other therapy?
Final question	Is there anything else? What is more?

Because the transcripts were vast and a coding process was essential to the results, the analysis was accomplished by systematic text condensation [10, 14, 15]. This includes: (a) reading the material several times to obtain an overall impression and to bracket previous preconceptions; (b) identifying units of meaning, representing different aspects of participants’ insistence on the course associated with acupuncture, and coding these; (c) condensing and summarizing the contents of each of the coded groups; and (d) generalizing the descriptions and concepts concerning the preservation of acupuncture. There were no repeat interviews and no transcripts were returned to the participants. To enhance the trustworthiness of the content, the coding was continually revisited and refined by the authors jointly. The data visualization software NVivo 8 (QSR International, Doncaster, Australia) was used to monitor this process throughout.

## Results

### Participants

There was a 4-month interval between the end of treatment and the study onset for one participant and a 2-month interval for three participants. The remaining participants (11) were still undergoing the therapy as of June 2014. The mean age was 32 years, and their occupations varied from office clerk, to merchant, teacher and civil servant. The mean treatment length was 4.25 months. Two participants became pregnant and thus did not continue the treatment, nine participants described positive bodily changes in addition to improvement in their lower abdomen pain, seven participants recovered their menstruation cycle, and two patients felt worse at times.

### Themes

There were 67 codes, 19 sub-themes and four overarching themes in the participants’ accounts relating to their insistence on lengthy but important acupuncture courses. The four overarching themes were: (1) the patients’ characteristics, including pregnancy aspiration and the fear of serious gynecological disease; (2) the patient–practitioner relationship, including the acupuncturist’s attitude towards the patients and the explanation of the disease from a TCM perspective; (3) the characteristics of acupuncture, such as the diversity of treatment modes, the synthetical effect, and the lack of side effects; and (4) the clinical environment, including exchanges of experiences between patients and a well-equipped setting.

### Patients’ characteristics

These generally refer to patient condition, illness beliefs, anxiety and adherence to the treatment course.

#### Pregnancy aspirations

Ten of the women with chronic PID had pregnancy-related concerns. Following traditional ideas, they felt significant responsibilities relating to childbearing as a necessary part of family life. Because chronic PID can lead to infertility, these women insisted on acupuncture treatment.

“I am afraid that I would lose the ability to have babies. My husband has always loved children. Without fertility, I think my life is incomplete. After deliberating with my family, they all support me…” (C3)

“After being married for 3 years, I am confused that I have never been pregnant from a normal sex life without contraception. I did not know the reason until I was checked by the gynecologist and diagnosed with chronic PID… of course, I will come here for improvement…” (C6)

As typical examples of patients in the reproductive age category, they put the needs of pregnancy and children first. Acupuncture gave these participants hope of recovery from PID, and they believed there would be few or no side effects, as they had been informed by their friends and relatives.

#### Fear of serious gynecological disease

Chronic PID may lead to increased risk of some serious gynecological diseases, such as ectopic pregnancy and even ovarian tumors. Perimenopausal women may have limited knowledge of chronic diseases, and thus may be anxious about their bodies and concerned about health care. Acupuncture can help meet their requirements in terms of integrated effects.

“I prefer TCM therapy, particularly acupuncture. As you know, I’m already more than 40 years old with a complex physical condition and am in fear of developing serious gynecological disease by chronic sickness…” (C8)

The perimenopausal period is an important time for women, and they should undergo physical examinations annually because of the associated health concerns. They are likely to choose acupuncture because of its advantages.

### The patient–practitioner relationship

The patient–practitioner relationship refers to suggestion, reassurance, and compassion during the communication between patients and practitioners.

#### The acupuncturist’s attitude towards patients

All participants mentioned the acupuncturist’s attitude towards them. They said that her soft voice, concerned eyes and gentle movement made them comfortable and relaxed, as if she was consoling uneasy emotions. Establishing such a rapport would tend to promote patients insisting on treatment.

“While suffering from chronic pelvic pain for 2 years, I have been extremely anxious. Doctor Gong is an acupuncturist who practices medical ethics. She values me and takes into account what I tell her about my symptoms and feelings…” (C2)

“I like…her attitude towards patients, although the effect has not reached my expectation to a certain extent…” (C7)

The acupuncturist’s attitude towards participants was an essential factor in their insistence on treatment. Some considered attitude to be even more vital than medical skill. Receiving concern and care from the acupuncturist gave the participants hope and confidence against their illness.

#### Explanation of the disease from a TCM perspective

The patients all wanted more details about their condition, but in most cases the acupuncturist was too busy to explain. To resolve this, an intern trained by the acupuncturist was responsible for recounting the details, particularly the model of TCM perception focusing on the integral condition.

“It sounds a bit like magic while they tell me about my condition in the model of TCM. For example, the etiology and mechanism of chronic PID are the cold-damp stagnation of the pelvis, and they try their best to give me an in-depth understanding of my condition…” (C5)

The patients generally lacked detailed knowledge about their illness and things they could do to help themselves. An explanation of the disease from a TCM perspective helps them better understand TCM and follow the doctor’s advice.

### The characteristics of acupuncture

These refer to the practice, effect, price and course of acupuncture.

#### The diversification of treatment modes

Acupuncture encompasses multiple therapies including needling, moxibustion, massage, cupping, and auriculotherapy. The individualization of treatment based on syndrome differentiation is one feature of TCM treatment, and there are diverse combinations of therapeutic techniques.

“Before treatment, the doctor told me that needling combined with moxibustion was best for my condition, so I tried it. After about three treatments I felt better than before, and indeed I did feel a sensation of chill in my abdomen two months ago (caused by cold-dampness in concept of TCM)…” (C11)

Diverse treatment modes mean the best possible outcome, and effective measures should be taken to remedy complicated chronic illness.

#### Synthetic effect and no side effects

TCM is a holistic therapy. Based on TCM theory, the outcome is not only improvement of the main symptoms but also addressing associated symptoms. Some women found associated symptoms to be improved beyond expectation during the treatment.

“Actually the intensity of pelvic pain is the same as ever, but I found that my sleep and appetite improved. At that time, it gave me confidence to keep on…” (C9)

Acupuncture has been shown over time to be free of adverse side effects, and this was a consideration in participants’ considering it as important for improving prenatal and postnatal care.

“It has no side effects compared with Western treatments to my knowledge, so I am ready for pregnancy and I hope the treatment is safe…” (C5)

### The clinical environment

This refers to the relationship of patients attending the same clinic, the health-care setting, distances involved and affiliated services.

#### The exchange of experience

Approximately 60% of the patients in the clinic have a gynecological or related illness. Participants responded that they prefer a group acupuncture environment. In such an environment they can exchange experiences regarding their physical condition, acupuncture, and improvement or aggravation of symptoms, and thus establish a peer relationship against the disease.

“I have no knowledge of my disease and that makes me worried. In the clinic I often meet the same people with chronic PID, we talk together and communicate the process of disease and…sometimes I will inform my other friends…” (C12)

“Communicating with [other patients] is pleasant…when someone tells me that she has become better, I think I will be better soon. Meanwhile, it seems that my abdominal pain is relieved…” (C10)

“Meeting the same people” in the clinic seems to have a mental placebo effect for these patients. The mutual support gained from each other is a complementary benefit to visiting the doctor.

#### Well-equipped setting

The clinic is large enough to accommodate 10 to 15 patients and has 10 beds and five chairs. There is sufficient equipment on hand, such as acupuncture needles, glass jars, plum-blossom needles, and moxibustion sticks.

“This place is clean, large, and well-equipped, so I feel comfortable. It is a formal medical facility. I have only been treated in a small clinic before, which doesn’t have single-use needles…” (C10)

Female participants with only a high-school education expressed that they valued this clinical environment, and that it added to their impression of the clinic’s credibility.

## Discussion

### Key findings

In this interview-based study, the results reveal a variety of reasons why the patients insist on a long-term and frequent acupuncture course. This is the first study to investigate such reasons among women with chronic PID. The key findings include the patient’s characteristics, the patient–practitioner relationship, the characteristics of acupuncture, and the clinical environment. All of the findings pointed to the outcome of acupuncture as effective in genuinely improving illness, and not just a placebo. Relevant research confirming this has been conducted [16, 17].

### Strengths and limitations

A qualitative method is appropriate for investigating why the participants might insist on treatment. Analysis was accomplished by systematic text condensation that allows the themes to develop naturally from the informants’ own voices, and empowers the informants to freely share opinions. Because they were given sufficient time, the participants elaborated and focused on the questions that they adequately understood. Some considered acupuncture to be ineffective in improving their main symptoms: this contributed to data acceptability and credibility.

Treatment lengths ranged from 3 to 7 months; hence, there is possible recall bias in clearly remembering the reasons in different degrees of care. Iterative questioning and adequate interview time were necessary to reduce bias that may have affected the results. The small number of interviews was a limitation of this study. However, it was necessary to use a small sample who met the criteria so as to ensure the quality of the study and reduce recall bias. Furthermore, the qualitative interview data in this study are all from one acupuncturist’s clinic, but strictly in the light of the analysis method, the individual differences were taken on the outcome.

### Comparison with other studies

To ensure a long-term effective acupuncture outcome, women with chronic PID should insist on a minimum of 3 months of therapy. This study shows four overarching reasons for this. Previous studies focused on the experience or acceptability of acupuncture, both of which are closely related to the preservation of the acupuncture process. Patients’ insistence on acupuncture treatment, and their compliance or willingness to undergo the course of treatment, are predicated on their finding it acceptable. It was reported that most patients with lower back pain who visit physicians do not clearly understand acupuncture. However, patients with lower back pain who receive recommendations from medical staff or friends are more likely to select acupuncture [18]. Additionally, this study shows that the exchange of experience and the explanation of disease from a TCM perspective contributed to patients’ insisting on treatment. With knowledge and understanding about the relationship between acupuncture and the disease, patients would be more likely to select acupuncture. In a survey investigating whether stroke patients would consider acupuncture as an option in their rehabilitation, most respondents (98%) wanted further information on acupuncture in stroke rehabilitation and 87% would consider acupuncture as an option [19].

Diverse treatment modes are essential to enhance acceptance of acupuncture. TCM originated from ancient Chinese philosophy, and is bound to treatment based on syndrome differentiation. Although it has developed rapidly worldwide, acupuncture research methods must meet international standards, which constrains the development of traditional techniques. The participants in this study described different experiences of individual treatment: undergoing holistic treatment, following the practitioner’s advice on lifestyle changes, and accompanying long-term benefits. A recent multicenter randomized controlled trial comparing the effects of individualized treatment, standard treatment, sham treatment, and non-acupuncture use in the treatment of knee osteoarthritis showed that individual acupuncture showed the highest efficacy, and its widespread use was encouraged [20].

### Specific effect and nonspecific effect

Specific effects are clearly defined outcomes caused by acupuncture. Nonspecific effects, however, are considered similar to a placebo effect, and are taken by some as refuting the real effects of acupuncture, and are a hindrance to its wider acceptance. Some participants in this study continued the course of treatment course even after they did not experience a specific effect. The reason for this is that the patient–practitioner relationship gave the participants hope in relation to their disease treatment, similar to a placebo effect. However, acupuncture was the primary medical technique for over 2000 years in China. The fact that TCM principles are so deeply ingrained in Chinese culture and psychology make it difficult to distinguish specific and nonspecific effects. Both of these effects support the other and thus achieve the best results; this holistic approach is a prominent feature of TCM. Further empirical studies are needed to illustrate this to the scientific world.

### Implications for practice and future research

During an acupuncture treatment course, the participants reported feeling hope, confidence and a sense of responsibility for their treatment, feelings that were related to the four overarching study themes. Chronic disease and ineffective treatment can be heavy psychological and physiological burdens for patients. Emotional support encourages patients to insist on treatment and to maintain improvements brought about by it. A previous study suggested that patients treated with acupuncture continued to experience improvements for up to 24 months after entering the trial [21]. Other acupuncture trials show similar long-term benefits [22, 23]. Whether acupuncture can alleviate chronic PID symptoms is largely dependent on the acupuncturist’s ability. The implications for practice are that acupuncturists should be equipped with medical knowledge and communication skills, and should facilitate patients’ active involvement in self-care and recovery. Future research on acupuncture should include improvements in qualitative studies, more extensive interviews, less bias, and a broader sample group.

## Conclusions

There are a variety of reasons why chronic PID patients persevere with acupuncture treatments. Specific and non-specific effects are distinct but are not divisible. Knowledge and understanding about the relationship between acupuncture and their disease encourage patients to choose acupuncture. Acupuncture should be widely developed and promoted while maintaining its characteristics. Participants reported feeling hope, confidence and a sense of responsibility for their treatment during the process, although the treatment outcomes were not always as expected. These feelings encourage patients to insist on treatment and maintain the improvements. These will contribute to an improved standing of acupuncture in the medical profession, and will enhance public attitudes towards acupuncture. However, this will require long-term concerted efforts by acupuncturists.

## Authors’ information

LYD is a master of acupuncture in Guangzhou University of Chinese Medicine. Her research is mainly in acupuncture for gynecology. GDF with a Ph.D. in acupuncture is a skillful clinical acupuncturist with license for more than 30 years and adept at the treatment of gynecological diseases.

## References

[1] Rasmussen CB, Faber MT, Jensen A, Høgdall E, Høgdall C, Blaakær J, Kjaer SK (2013). Pelvic inflammatory disease and risk of invasive ovarian cancer and ovarian borderline tumors. Cancer Causes Control.

[2] Ozel S, Arslan H, Tufan ZK, Uzunkulaoğlu T, Akarsu D, Seven A (2011). Acupuncture in the treatment of chronic pelvic pain secondary to pelvic inflammatory disease. Acupunct Med.

[3] Xin X, Xu Q, Gong D (2005). Treatment of chronic pelvic inflammation with acupuncture and TDP irradiation–a report of 23 cases. J Tradit Chin Med.

[4] Qu F, Zhou J, Burrows E, Luo Q, Wu Y, Wang T, Huang H (2008). Acupuncture may improve the absorption of baicalin from the extracts of Scutellaria baicalensis Georgi in rats with pelvic inflammation. Forsch Komplementmed.

[5] Zhen HL, Wang Y, Liu XJ (2008). Observation on therapeutic effect of warming needle moxibustion on chronic pelvic inflammation of cold-damp stagnation type. Zhongguo Zhen Jiu.

[6] Wang QC, Chen YM, Jia MJ, Zhai HL (2012). Efficacy observation of chronic pelvic inflammation of different differentiated patterns/syndromes treated with acupoint embedding therapy. Zhongguo Zhen Jiu.

[7] Feng Z (2014). Observation on chronic pelvic inflammation treated by acupuncture of 25 cases. Yunnan J Tradit Chin Med Mater Med.

[8] Woźniak PR, Stachowiak GP, Pieta-Dolińska AK, Oszukowski PJ (2003). Anti-phlogistic and immunocompetent effects of aupuncture treatment in women suffering from chronic pelvic inflammatory diseases. Am J Chin Med.

[9] Hopton A, Thomas K, MacPherson H (2013). The acceptability of acupuncture for low back pain: a qualitative study of patient’s experiences nested within a randomised controlled trial. PLoS One.

[10] Alraek T, Malterud K (2009). Acupuncture for menopausal hot flashes: a qualitative study about patient experiences. J Altern Complement Med.

[11] Billhult A, Stener-Victorin E (2012). Acupuncture with manual and low frequency electrical stimulation as experienced by women with polycystic ovary syndrome: a qualitative study. BMC Complement Altern Med.

[12] Asprey A, Paterson C, White A (2012). ‘All in the same boat’: a qualitative study of patients’ attitudes and experiences in group acupuncture clinics. Acupunct Med.

[13] Bishop FL, Lewith GT (2013). Patients’ preconceptions of acupuncture: a qualitative study exploring the decisions patients make when seeking acupuncture. BMC Complement Altern Med.

[14] Giorgi A, Giorgi A (1985). Sketch of a Psychological Phenomenological Method. Phenomenology and Psychological Research.

[15] Malterud K (2001). Qualitative research: standards, challenges, and guidelines. Lancet.

[16] Karner M, Brazkiewicz F, Remppis A, Fischer J, Gerlach O, Stremmel W, Subramanian SV, Greten HJ (2013). Objectifying specific and nonspecific effects of acupuncture: a double-blinded randomised trial in osteoarthritis of the knee. Evid Based Complement Alternat Med.

[17] Paterson C, Dieppe P (2005). Characteristic and incidental (placebo) effects in complex interventions such as acupuncture. Br Med J.

[18] Zhang Y, Cheng LJ (2010). Acupuncture or physical therapy? Low-back pain patients’ choice-a qualitative study in a general hospital. China Value in Health.

[19] Yam W, Wilkinson JM (2010). Is acupuncture an acceptable option in stroke rehabilitation? A survey of stroke patients. Complement Ther Med.

[20] Kim EJ, Lim CY, Lee EY, Lee SD, Kim KS (2013). Comparing the effects of individualized, standard, sham and no acupuncture in the treatment of knee osteoarthritis: a multicenter randomized controlled trial. Trials.

[21] Thomas KJ, MacPherson H, Ratcliffe J, Thorpe L, Brazier J, Campbell M, Fitter M, Roman M, Walters S, Nicholl JP (2005). Longer term clinical and economic benefits of offering acupuncture care to patients with chronic low back pain. Health Technol Assess.

[22] Vickers AJ, Rees RW, Zollman CE, McCarney R, Smith CM, Ellis N, Fisher P, Van Haselen R, Wonderling D, Grieve R (2004). Acupuncture of chronic headache disorders in primary care: randomised controlled trial and economic analysis. Health Technol Assess.

[23] Kjendahl A, Sallstrom S, Osten PE, Stanghelle JK, Borchgrevink CF (1997). A one year follow-up study on the effects of acupuncture in the treatment of stroke patients in the subacute stage: a randomized, controlled study. Clin Rehabil.

[CR24] The pre-publication history for this paper can be accessed here:http://www.biomedcentral.com/1472-6882/14/345/prepub

